# Correction: Salwén et al. Regulatory T Cells and IFNγ in Mercury-Induced Autoimmunity: Insights from Adoptive Transfer in B10.S Mice. Biology 2026, 15, 298

**DOI:** 10.3390/biology15141139

**Published:** 2026-07-13

**Authors:** Rebecka Salwén, Mehdi Amirhosseini, Said Havarinasab

**Affiliations:** 1 Division of Inflammation and Infection (II), Department of Biomedical and Clinical Sciences, Linköping University, 581 83 Linkoping, Sweden; 2 Division of Clinical Chemistry and Pharmacology (KKF), Department of Biomedical and Clinical Sciences, Linköping University, 581 83 Linkoping, Sweden

## Error in Figure

In the original publication [1], there was a mistake in Figure 2A as published. The image currently displayed in this figure does not originate from a Hg-treated B10.S donor, as stated in the figure legend. The corrected Figure 2A is now replaced by the correct image taken from a Hg-treated B10.S donor mouse and it appears below. The authors state that the scientific conclusions are unaffected. This correction was approved by the Academic Editor. The original publication has also been updated.

## Figures and Tables

**Figure 2:**
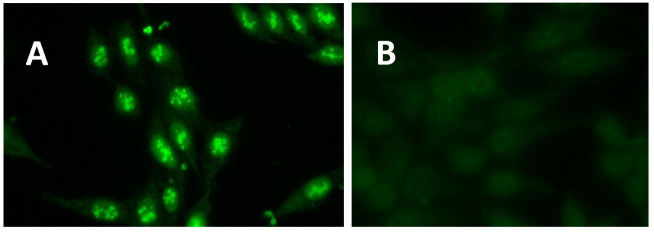
Serum antinucleolar antibodies (ANoA), exhibiting a distinct clumpy nucleolar staining pattern, were assessed via indirect immunofluorescence in mercury-treated B10.S donor mice (**A**). In contrast, no nucleolar staining of HEp-2 cells was observed when using serum from mercury-treated B10.S IFNγ−/− donor mice (**B**). The images depict animals treated with mercury for 4 weeks. Serum samples were incubated with HEp-2 cells, and staining was detected using FITC-conjugated anti-mouse IgG antibodies.
